# Blueberry-Derived Exosome-Like Nanoparticles Counter the Response to TNF-α-Induced Change on Gene Expression in EA.hy926 Cells

**DOI:** 10.3390/biom10050742

**Published:** 2020-05-10

**Authors:** Mariangela De Robertis, Angelo Sarra, Valentina D’Oria, Francesco Mura, Federico Bordi, Paolo Postorino, Deborah Fratantonio

**Affiliations:** 1 Institute of Biomembranes, Bioenergetics and Molecular Biotechnologies, Consiglio Nazionale delle Ricerche (CNR), 70126 Bari, Italy; m.derobertis@ibiom.cnr.it; 2 Department of Biosciences, Biotechnology and Biopharmaceutics, University of Bari Aldo Moro, 70125 Bari, Italy; 3 Department of Science, University of Roma Tre, 00145 Rome, Italy; angelo.sarra@roma1.infn.it; 4 CNR-ISC UOS Sapienza, Sapienza University of Rome, 00185 Rome, Italy; federico.bordi@roma1.infn.it; 5 Bambino Gesù Children’s Hospital IRCCS, Research Laboratories, 00146 Rome, Italy; valentina.doria@opbg.net; 6 Center for Nanotechnology for Engineering (CNIS), Sapienza University of Rome, 00185 Rome, Italy; francesco.mura@uniroma1.it; 7 Department of Physics, Sapienza University of Rome, 00185 Rome, Italy; paolo.postorino@roma1.infn.it

**Keywords:** blueberry, cross-kingdom, exosome-like nanoparticles, gene expression, inflammation, oxidative stress, pathway analysis, uptake

## Abstract

Exosome-like nanoparticles (ELNs) are attracting interest as important vehicles of intercellular communication, both in prokaryotes and eukaryotes. Recently, dietary nanoparticles similar to mammalian exosomes have attracted attention for these features. In particular they appear to be relevant in the modulation of several cellular processes as well as candidate carriers of bioactive molecules (proteins, lipids, and nucleic acids, including miRNAs) with therapeutic value. Herein, we investigated the cellular uptake of blueberry-derived ELNs (B-ELNs) by a human stabilized endothelial cell line (EA.hy926) and the ability of B-ELNs to modulate the expression of inflammatory genes as the response of tumor necrosis factor-α (TNF-α). Our results indicate that 1) EA.hy926 cells internalize B-ELNs in a dose-dependent manner; 2) pretreatment with B-ELNs counters TNF-α-induced reactive oxygen species (ROS) generation and loss of cell viability and modulates the differential expression of 29 genes (fold change > 1.5) induced by TNF-α compared to control; 3) pathway analysis reveals their involvement in a total of 340 canonical pathways, 121 KEGG pathways, and 121 GO Biological processes; and 4) the intersection between differentially expressed (DE) genes and miRNAs contained in B-ELNs unveils a set of candidate target genes, such as prostaglandin I2 synthase (PTGIS), mitogen-activated protein kinase 14 (MAPK14), and phosphodiesterase 7A (PDE7A), for ELNs-contained cargo. In conclusion, our study indicates that B-ELNs can be considered candidate therapeutic carriers of bioactive compounds potentially able to protect vascular system against various stressors.

## 1. Introduction

Cell-to-cell communication is a physiological mechanism that contributes in maintaining tissue functions and homeostasis. In the last decade, we have assisted an increased interest in cell-to-cell interaction mediated by extracellular vesicles (EVs), thanks to their ability to carry a variety of molecules from the producing cell to target cells [1]. EVs are membranous vesicles of different size, usually classified into different types according to their dimension [2]. Among them, exosomes are nanometer-sized vesicles in a range of 30–100 nm, present in many biological fluids of different organisms [3]. Previous studies have identified and reported the existence of exosome-like nanoparticles (ELNs) in plants [4], fruits [5], and very recently also in mushrooms [6].

ELNs assumed with dietary intake carry a cargo of proteins, lipids, mRNAs, and/or miRNAs [1,7,8], that can be transferred to the consuming organisms and then to the recipient cells, acting as extracellular messengers. Therefore, ELNs can mediate a specific “trans-kingdom” cell and tissue response [9,10]. Supporting data coming from independent laboratory indicate that nanoparticles derived from edible plants such as grape and grapefruit [5,11], ginger [12], and carrots [13] have anti-inflammatory properties [14]; ELNs from ginger inhibits inflammasome activation [12]; citrus-lemon ELNs demonstrate anti-oxidant properties and antitumor effect [15,16,17]; and apple-derived nanoparticles could affect intestinal transporters [18]. All these findings open up a new perspectives on the therapeutic potential of natural/food compounds [14,19,20].

Although it has been widely demonstrated the ability of compounds and/or specific extracts from different plants in modulating vascular cell response against agents inducing endothelial dysfunction [21,22,23], the specific role of ELNs in vascular health and disease is still largely unknown, therefore vascular endothelial cells may represent a specific target for exogenous ELNs. Endothelial cells treated with TNF-α represent a widely-used model to investigate the effects of natural compounds to modulate endothelial dysfunction and inflammation [24].

Here, we identified and characterized ELNs from *Vaccinium ashei* (family Ericaceae), commonly known as blueberries (BB), as a new food component able to affect endothelial cell response to TNF-α and to modulate the expression of genes involved in inflammatory response, cytokine release, and oxidative stress. In addition, by combining our data with a published library of miRNAs contained in B-ELNs, we identified a limited set of candidate mRNA targets.

## 2. Materials and Methods

### 2.1. Isolation and Characterization of B-ELNs

*V. ashei* fruits were purchased at local grocery, supplied by an Italian company (Aurora fruit, Verona, Italia). BB were washed three times in water and processed always between the third and sixth day after packaging. 125 g of fresh fruits were squeezed to obtain approximately 80 mL of juice. BB juice was analyzed and characterized for its anthocyanin content using a spectrophotometer (UV-1700 Spectrophotometer, Shimadzu, Kyoto, Japan). Anthocyanins, a group of natural compounds used as an internal control, have two absorbance peaks: One at A280 nm in the UV range and another one at 520 nm in visible range. In particular, it was compared with cyanidin-3-glucoside (C3G), one of the most common anthocyanin present in nature [25].

BB juice was diluted 1:5 with phosphate buffered saline solution (PBS), passed through a metallic strainer and sequentially centrifuged at 1000× *g* for 10 min, 5000× *g* for 20 min and 1.1 × 10^4^× *g* for 50 min. The supernatant was filtered at 0.45 μm and ultra-filtered in 10,000 MWCO membrane (Sartorius) using peristaltic pomp (Watson Marlow 520S, Falmouth, UK) at 125 g/min. Concentrated juice was then filtered at 0.22 μm and then centrifuged at 1.2 × 10^5^× g for 90 min in a SW41Ti rotor using OptimaTM XPN-100 Ultracentrifuge, Beckman Coulter, SW 40 Ti-rotor (Beckman, USA). B-ELNs protein content was measured using a BCA protein assay kit (Thermo Scientific Pierce, Rockford, IL, USA) and was stored at −80 °C before use. For each experiment, B-ELNs pellet was freshly rinsed with sterile PBS.

Dynamic light scattering (DLS) measurements were performed employing a MALVERN Nano Zetasizer apparatus equipped with a 5 mW HeNe laser (Malvern Instruments LTD, Worcestershire, UK). This system uses backscatter detection, i.e., the scattered light is collected at 173°. To obtain the size distribution, the measured autocorrelation functions were analyzed using the CONTIN algorithm [26]. Decay times are used to determine the distribution of the diffusion coefficients D_0_ of the particles, which in turn can be converted in a distribution of apparent hydrodynamic diameter, D_h_, using the Stokes–Einstein relationship D_h_ = k_B_T /3πηD_0_, where k_B_ is the Boltzmann constant, T the absolute temperature and η the solvent viscosity. Measurements were performed at fixed temperature of 25 °C assuming the solvent viscosity was that of water. The values of the diameter shown in this work are obtained from intensity weighted distributions [26,27].

Laser transmission spectroscopy (LTS) technique was used to obtain the density distribution of colloidal suspensions like our B-ELNs samples. A big advantage of LTS over the more traditional diffusion techniques, such as DLS and Nanoparticle Tracking Analysis (NTA), is that this method furnishes the distribution of the geometrical sizes (and in principle the shape) of the suspended particles and not their equivalent “hydrodynamic” radii (radius of a sphere with the same diffusion coefficient), and moreover their absolute concentration in the suspension (not the relative weight of the different size classes). The LTS technique measures the transmittance of a laser beam through the aqueous suspension of vesicles as a function of the wavelength [28,29]. Given the transmission coefficient T(λ) and the known wavelength-size dependent properties, i.e., Mie scattering cross-section σ(λ,r) of the vesicles, represented in the present case as shelled spheres [30], the particle density distribution n(r) as a function of their size can be calculated through the Beer–Lambert law [29]. Transmission data are analyzed and inverted by using a mean square root-based algorithm giving the particle size distribution in terms of their absolute concentration. The integral of the density distribution provides the total number of vesicles per milliliter of solution.

Images of B-ELNs were taken using scanning electron microscopy (SEM) (Auriga 405, Zeiss, Oberkochen, Germany. Briefly, fresh rinsed B-ELNs were fixed with 2.5% glutaraldehyde in 0.15 M cacodylate buffer (pH 7.2) at room temperature. Field emission scanning Electron microscopy (FESEM) analysis was performed using a Zeiss Auriga microscope, operating at a low acceleration voltage and current in order to avoid beam damage of vesicles.

### 2.2. EA.hy926 Cell Culture and Experimental Design

The human stabilized endothelial EA.hy926 cell line was kindly provided by Dr. Del Fattore A. (Bambino Gesù Children’s Hospital, Rome, Italy). Cells were cultured in DMEM with 5.5 mM glucose (EuroClone, Italy) supplemented with 10% inactivated and exosomes-depleted fetal bovine serum (FBS), 2 mM L-Glutamine, 100 IU/mL penicillin, 0.1 mg/mL streptomycin. Cells were maintained at 37 °C in 5% CO_2_. For all the experiments B-ELNs pellet was freshly dissolved in PBS and then diluted in culture media to obtain the desired final concentration. B-ELNs concentration range was chosen on the basis of previous studies focused on the effect of ELNs [11,16]. Subconfluent cells were pretreated with B-ELNs according to each experimental condition as described below. At the end of incubation, cells were washed with PBS under sterile conditions and then incubated for 2 h with TNF-α 20 ng/mL as previously reported by Fratantonio et al. [24]. Untreated cells, exposed only to the vehicle (PBS) were used as a control.

### 2.3. B-ELNs Cellular Transport and Time Course Study

B-ELNs isolated as reported above were labeled using PKH26 Red Fluorescent Cell Linker Kit (Sigma-Aldrich, St. Louis, MO, USA), according to the manufacturer’s protocol with some modifications. Briefly, B-ELNs pellet was suspended in 1 mL Diluent C. Separately, and 1 mL Diluent C was mixed with 4 μL fluoraphore-PKH26 (stain solution). The B-ELNs suspension was mixed with the stain solution and incubated for 4 min. The labeling reaction was immediately washed with PBS to remove the unlabeled dye using 10K Amicon^®^ Ultra-0.5 mL centrifugal filter (Millipore, Darmstadt, Germany) by centrifugation according to manufacturer’s instructions.

In a typical experiment, 4 × 10^4^ EA.hy926 cells/well were seeded in a 24-well plate and allowed to adhere for 24 h, 75% confluent. Freshly PHK26-labeled B-ELNs were added to each well at the desired final concentration. To evaluate a dose–response effect cells were incubated with 10, 20, and 40 µg/mL for 3 h. In the time course study 1 × 10^4^ EA.hy926 cells/well were seeded in a 96-well plate and allowed to adhere for 24 h, 75% confluent and only 20 µg/mL concentration was tested at 0.5, 1, 3, and 6 h. Media were removed and cells were washed three times with sterile PBS to remove excess of vesicles. Controls were prepared by washing the cells immediately after addition and removal of PHK26-labeled B-ELNs. Cell fluorescence was measured in a microplate fluorescence detector (BioTek™ Synergy™ H1, Vermont, USA). Cells were harvested using trypsin and counted using a hemocytometer (Z359629, Merk, Darmstadt, Germany). Units of fluorescence were converted into mass of B-ELNs bound by labeling a known mass of B-ELNs (protein) with fluorophore, and quantifying the fluorescence after removing unbound fluorophore. Fluorescence readings were corrected for cell auto fluorescence by subtracting signals measured in cells incubated with exosome-depleted media.

B-ELNs uptake was also evaluated by Leica TCS-SP8X laser-scanning confocal microscope (Leica Microsystems, Mannheim, Germany) equipped with tunable white light laser (WLL) source, 405 nm diode laser, 3 Internal Spectral Detector Channels (PMT) and 2 Internal Spectral Detector Channels (HyD) GaAsP. In this case, cells were grown on glass slide chamber (Falcon) and lead to adhere for 24 h. Cell were treated with 20 and 40 µg/mL of labeled vesicles for 3 h at 37 °C. Cells were stained with Wheat Germ Agglutinin 488 (WGA) (Invitrogen™) for 10 min, washed two times with cold PBS and fixed with 4% paraformaldehyde solution for 10 minutes at room temperature. After a washing step with PBS, nuclei were stained by incubating cells with a Hoechst 33258 (Invitrogen™) in PBS solution (1:5000) for 10 minutes. Fixed cells were mounted with a glycerol/PBS solution (3:1) and kept covered to prevent dye photo-bleaching before fluorescence image acquisition. Sequential confocal images were acquired using a HC PL APO 60x oil-immersion objective (1.40 numerical aperture, NA, Leica Microsystems, Mannheim, Germany) with a 1024 × 1024 format, scan speed 600 Hz, and z-step size of 0.50 µm.

### 2.4. Cell Viability

The possible cytotoxic effect of the B-ELNs and the viability after TNF-α treatment on the EA.hy926 cell line was investigated using Sulforhodamine B, a dye that binds to cellular proteins, as previously described [31]. Briefly, cells were seeded at density 1 x 10^4^ in 96-well plate and incubated overnight at 37 °C and 5% CO_2_. Cells were then pretreated with 20 and 40 µg/mL of B-ELNs for 3 h, and then exposed to TNF-α 20 ng/mL for 2 h. Untreated cells, exposed to PBS only were used as control. The absorbance was measured at a wavelength of 565 nm using a Shimadzu UV-1601 spectrophotometer (Shimadzu, Japan).

### 2.5. Detection of Intracellular Superoxide Anion/Superoxide-Derived ROS with Dihydroethidium (DHE)

Intracellular superoxide anion/superoxide-derived ROS was monitored using the fluorescent probe dihydroethidium. EA.hy926 cells were grown on glass coverslips and incubated with a fresh working solution containing 5 µM DHE (Sigma) in sterile conditions for 30 min at 37 °C. After this incubation time cells were washed two times with PBS and a microplate reader was used to measure the fluorescence (Ex 480–520 nm; Em 570–600 nm) [32].

### 2.6. RNA Isolation, qRT-PCR, and Gene Expression Profile

Total RNA was isolated from cells pretreated with 20 µg/mL of B-ELNs for 3 h and then exposed or not to TNF-α 20 ng/mL for 2 h, using a commercial RNA purification kit (Direct-zol RNA MiniPrep, ZYMORESEARCH, Irvine, CA, USA) according to the manufacturer’s protocol. RNA quality and quantity was assessed by Agilent 2100 Bioanalyzer. One microgram of total RNA was reverse transcribed using High Capacity cDNA reverse transcription kit (Applied Biosystems, Foster City, CA, USA) according to the manufacturer’s protocol. Gene expression profiling was performed using pre-configured Taqman^®^ low-density array (TLDA) “Human Inflammation” cards (Applied Biosystems) to analyze 96 selected candidate genes of a broad range of inflammatory response (including four housekeeping genes). The analysis was carried out on a 12X QuantStudio fast RT-PCR using SDS 2.2 software (Applied Biosystems, Foster City, CA, USA) according to the manufacturer’s protocols. Briefly, 100 ng of single-stranded cDNA were combined to TaqMan Universal PCR Master Mix 2X (Applied Biosystems) and water was added to a final volume of 100 µl per port. Thermal cycling conditions were the following: 50 °C for 2 min, 95 °C for 10 min, 40 cycles at 95 °C for 15 s and 60 °C for 1 min. A TLDA Human Inflammation Panel was used to analyze 96 genes (including housekeeping). Then, interesting genes were validated in a single reaction assay using Taqman probe (Applied Biosystems). The list of the assay is provided in Supplemental Table S1. Relative gene expression was normalized using the geometric mean of two house-keeping genes (GUSB and ACTIN) and calculated using the ΔΔCt method [33].

### 2.7. Functional Enrichment Analysis

The biological interpretation of the low-density array data was conducted using Qiagen’s (Dusseldorf, Germany) Ingenuity Pathway Analysis version 7 (IPA7, http://www.ingenuity.com). The functional enrichment analysis of DE genes in cells exposed to TNF-α and in cells pretreated with B-ELNs before TNF-α exposure versus untreated cells was based on the prior calculation of the Z-scores, by which we inferred the activation states of the obtained biological functions. Based on the DE genes, enriched gene ontology (GO) and KEGG functional pathways were obtained using NetworkAnalyst 3.0 (networkanalyst.ca, USA) [34]. Statistical significance (Fisher’s exact test, “overlap *p*-value”) was calculated to measure the likelihood that an association between a set of genes and a related function was not due to the chance. We considered *p*-values < 0.05 and Z-scores > 0 (activation) or < 0 (inhibition).

### 2.8. Prediction and Functional Annotation of miRNA Target Genes

The putative target genes for miRNAs of interest retrieved by Xiao et al. [35] were predicted using the plant miRNA target prediction online software psRNATarget (bio.tool V2, 2017 release) (http://plantgrn.noble.org/psRNATarget/). We selected the V2 Scoring Schema (2017 release), and applied “Homo Sapiens (human), transcript, Human genomic sequencing project”. psRNATarget is a new web server designed to integrate and analyze reverse complementary matching between target transcripts and small RNAs [36]. The software also evaluates the target-site accessibility by calculating the unpaired energy utilized to unfold the secondary structure around the miRNA target site in the mRNA. The following default parameter settings were applied: Penalty for opening gap = 2, penalty for extending gap = 0.5, expectation = 5, penalty for GU pair = 1, penalty for other mismatches = 1, HSP size = 19 and seed region = 2–7 nucleotides.

### 2.9. Statistics

Statistical significance of differences among treatment groups was assessed using 1-factor ANOVA and Dunnetts t test for post hoc comparisons between treatment groups and control. Time course experiments were analyzed using linear regression analysis. Analyses were performed using Prism 5 statistical software (GraphPad Software Inc., San Diego, CA, USA). Differences were considered significant at *p* < 0.05. Results are presented as means ± Standard Deviation (SD) and represent independent biological replicates.

## 3. Results

### 3.1. Blueberry (Vaccinium Ashei) Contains Exosome-Like Nanoparticles

In order to take into account the batch-to-batch variability of BB juice we performed a spectrophotometric analysis to normalize juices according to their content of cyanidin-3-0-glucoside (C3G), based on its specific absorbance peak at 520 nm. Arbitrarily, B-ELNs were then isolated and characterized as described in methods section (Supplementary Figure S1), starting from BB juices containing 150.3 ± 32.6 µM of C3G equivalent (n = 5). The average yield for each isolation, in terms of proteins amount, was 1.28 ± 0.11 mg/mL (Supplemental Figure S2).

Size and particle concentration of B-ELNs were characterized by DLS and LTS analysis. In DLS analysis, the size distribution is weighted by the intensity of scattering, which overestimates the contribution of highest diameters, implying that a few large vesicles can have the same intensity of many small vesicles. On the contrary, LTS analysis provides a density distribution that depends directly by the number of vesicles per ml and allows the obtainment of the different populations of the sample with higher resolution.

**Figure 1:**
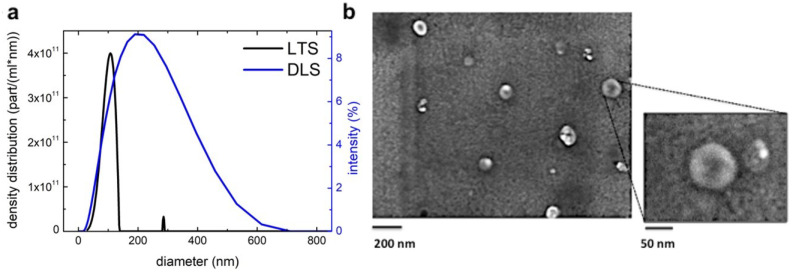
Characterization of blueberry-derived exosome-like nanoparticles (B-ELNs) (**a**) size distribution was determined by laser transmission spectroscopy (LTS) and dynamic light scattering (DLS) analysis. The black line shows results obtained with LTS apparatus and the blue line with DLS apparatus (**b**) morphology of B-ELNs was analyzed under scanning electron microscopy (SEM). The large-field was obtained at 70× magnification (scale bar 200 nm); the insert at 150× (scale bar 50 nm).

Although the two techniques provided different responses, the obtained results agreed with each other. In particular, as for DLS we obtained a single broad population (198 ± 112 nm of diameter), the LTS showed two populations with 108 ± 14 nm and 286 ± 6 nm of diameter are identified (the higher size population is probably due to aggregates). Moreover, by calculating the LTS density distributions reported in Figure 1a, the concentrations of vesicle populations resulted in the range of 2.2 ± 0.2 × 10^13^ vesicles/mL and 8.7 ± 0.9 × 10^10^ vesicles/mL, respectively, calculated as previously reported [37].

Scanning electron microscopy (SEM) analysis showed a heterogeneous population of vesicles with an average diameter of 114 ± 36 nm (n = 3) in agreement also with LTS results (Figure 1b).

### 3.2. B-ELNs are Internalized by EA.hy926 in a Dose-Depend Manner

**Figure 2:**
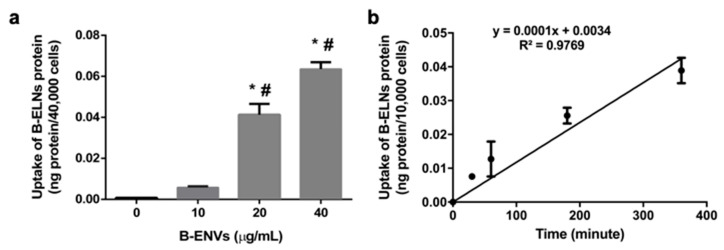
(**a**) B-ELNs uptake into human stabilized endothelial cell line (EA.hy926 cells) as a function of dose 0–40 µg/mL proteins at 37 °C for 3 h. (n = 3). (**b**) B-ELNs uptake into EA.hy926 cells as a function of time at a concentration of 20 μg/μL and a temperature of 37 °C (n = 3). Values are means ± SD of three separate experiments. *p < 0.05 vs. control, #0.05 vs. B-ELNs 10 μg/μL.

The first significantly detectable dose of B-ELNs internalized by EA.hy926 cells after 3 h exposure was 20 µg/mL (nanoparticles proteins) (Figure 2a). Therefore, all experiments related to the uptake and time course have been conducted using 20 and 40 µg/mL. We observed a linear time course of the cellular uptake up to 6 h at a concentration of 20 μg/mL (nanoparticles proteins), suggesting that at 3 h from the beginning of exposure the uptake was still in a liner range (Figure 2b). According to this observation, 3 h was chosen as end point for our observations. After this study, we elected to administer to cells a dose equal to 20 µg /mL.

**Figure 3:**
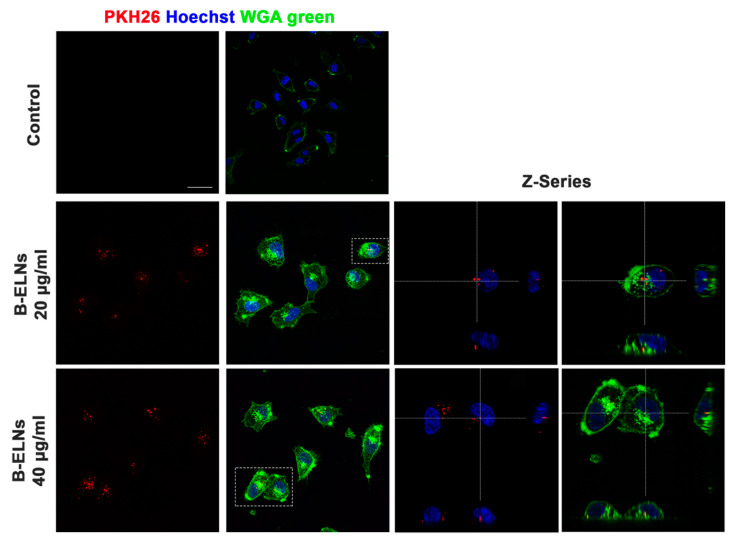
Analysis at confocal microscopy of EA.hy926 treated for 3 h with 20–40 µg/mL of B-ELNs proteins, compared with cells exposed to vehicle (Control). Cells membrane was stained with Wheat Germ Agglutinin 488 (WGA), nuclear staining was performed using Hoechst (blue), B-ELNs were labeled with PKH26 (red). Representative images were collected using 40X resolution (bar 50 µm) Z-series using 60× resolution (n = 3).

Confocal microscopy analysis further confirmed that B-ELNs were internalized rather than bound to the external cellular membrane and that the cellular uptake occurred in a dose-dependent manner (Figure 3).

### 3.3. B-ELNs Protect EA.hy926 from TNF-α-Induced Cytotoxicity and Oxidative Stress

**Figure 4:**
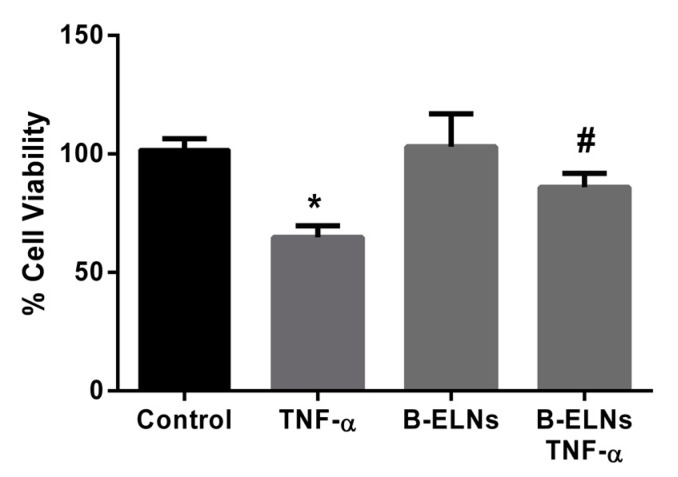
Viability of EA.hy926 cells (sulforhodamine B assay) after pretreatment with B-ELNs (20 µg/mL) for 3 h, and then exposed for 2 h to tumor necrosis factor-α (TNF-α) (20 ng/mL). Cell treated with the same volume of vehicle (PBS) were used as control. Data represent the percentages of viable cells compared to control and are expressed as mean ± SD of three separate experiments. *p < 0.05 vs. control; #*p* < 0.05 vs. cell exposed to TNF-α 20 ng/mL.

To investigate the effects of B-ELNs on the response of endothelial cells to TNF-α, EA.hy926 cells were pre-treated for 3 h with B-ELNs 20 µg/mL (nanoparticles proteins) then exposed to 20 ng/mL of TNF-α for 2 h. Concentrations of TNF-α and exposure time used in the present study was chosen according to previous studies published by our group and by other investigators [24]. The exposure to TNF-α induced significant cell damage leading to a decrease of cell viability. This effect was partially countered by the pretreatment with B-ELNs, while B-ELNs alone did not affect cell viability (Figure 4).

**Figure 5:**
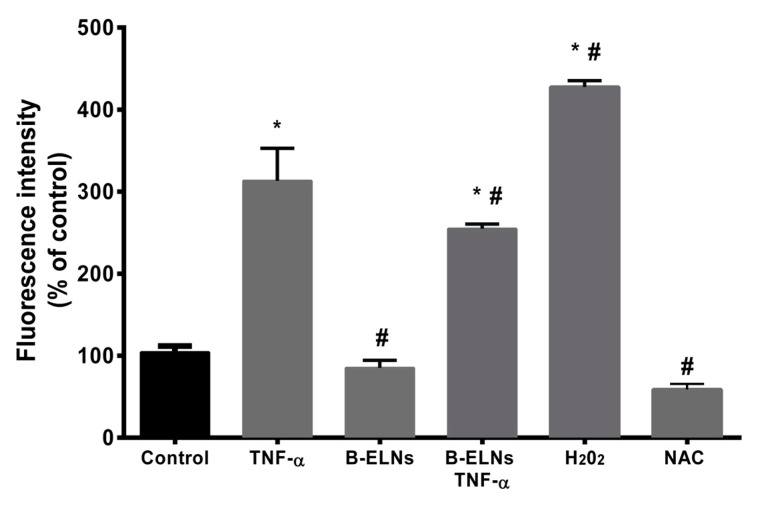
Intracellular reactive oxygen species (ROS) production in EA.hy926 cells pretreated with B-ELNs (20 μg/mL) for 3 h, and then exposed to TNF-α (20 ng/mL) for 2 h. Vehicle-treated cells were used as controls. Cells exposed to 50 µM of H_2_0_2_ for 1 h were used as positive control of increased intra-cellular ROS level, while cells treated with 200 µM N-acetyl-l-cysteine (NAC) as negative control. Results are expressed as relative ethidium fluorescent intensity (percentage of control), and are reported as mean ± SD of three separate experiments. *p < 0.05 vs. control; # *p* < 0.05 vs. control treated with TNF-α.

Moreover, in our experimental conditions, TNF-α treatment was associated to a significant increase of ROS production in comparison to control cells. The pre-treatment with B-ELNs was associated to a significant reduction of reactive species production. N-acetyl-l-cysteine (NAC), commonly used as a standard intracellular reducing agent, [37] and H_2_0_2_ were utilized as a negative and positive controls, respectively (Figure 5). Conversely, the treatment with B-ELNs 20 µg/mL alone was not associated with any changes of the intracellular level of ROS.

### 3.4. Effect of B-ELNs on TNF-α Induced mRNA Expression Changes

**Figure 6:**
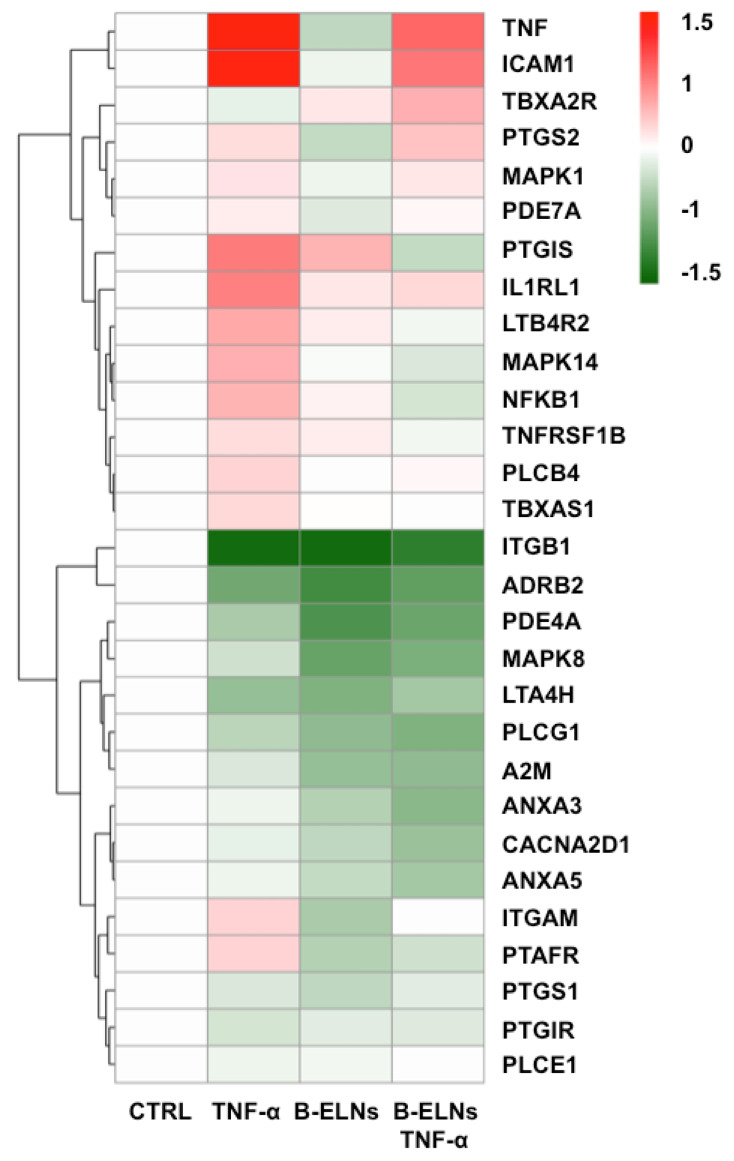
Heat map of the 29 differential expressed genes in EA.hy926 cells exposed to TNF-α (20 ng/mL) for 2 h and pretreated or not with B-ELNs (20 µg/mL) for 3 h. Data are represented as log fold change between −1.5 and +1.5 versus control.

A pre-configured TLDA “human inflammation” card was used to evaluate and to identify a set of genes significantly modulated by TNF-α in EA.hy926 cells and a possible effect of the pretreatment with B-ELNs. Out of a total 96 genes included in the panel, 29 genes were considered significantly modulated by TNF-α according to an arbitrary threshold > ±1.5 fold change. The results are summarized as a “heat map” in order to provide a visualization “at glance” of the overall effect of TNF-α on genes expression and of the modulation by B-ELNs (Figure 6).

We focused our attention on the modulatory effect of B-ELNs on a limited number of genes already known to be up-regulated by TNF-α in endothelial cells, namely: tumor necrosis factor, TNF; nuclear factor kappa-light-chain-enhancer of activated B cells, NF-kB; intercellular adhesion molecule 1, ICAM1 [22]; phosphodiesterase 7A, PDE7A [38]; mitogen-activated protein kinase 1 and 14, MAKP1 and MAPK14 [39]; prostaglandin I2 (prostacyclin) synthase, PTGIS [40]; interleukin 1 receptor-like 1, IL1RL1 [41], and Toll-like receptor, TLR [42].

In contrast, those genes whose modulation was not fully appreciated within the pre-treatment with B-ELNs and TNF-α compared to the TNF-α alone, were not deeply taken into account in the subsequent analysis.

**Figure 7:**
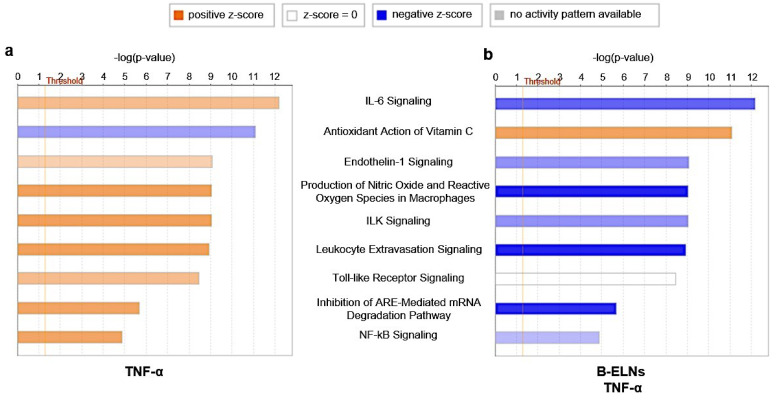
Enriched canonical pathways of 29 differentially expressed (DE) genes in EA.hy926 cells exposed to TNF-α (**a**) and pretreated with B-ELNs before TNF-α exposure (**b**). The significance of canonical pathways was determined by Ingenuity Pathway Analysis (IPA)’s default threshold [–log(*p*-value)]> 1.3. *p*-value was calculated by Fisher’s exact test. Color gradients of histograms vary from red (activation) to blue (inhibition) according to the Z-scores calculated for each enriched function.

Pathway analysis of DE genes, with a particular interest toward the significantly modulation by the pretreated with B-ELNs and TNF-α compared to TNF-α-treated cells, indicated their involvement in 340 canonical pathways (Supplementary Table S2). In Figure 7 we reported the canonical pathways specifically involved in vascular functions and inflammation response showing a modulation of TNF-α on the expression of IL-6 and ILK signaling, Endothelin-1 and Leukocytes extravasation signaling, TLR and NF-KB signaling and also against oxidative stress including antioxidant action. In particular, we observed a “positive z-score” (activation) of the pathways mentioned above under TNF-α treatment (Figure 7a). Noteworthy, the pretreatment with B-ELNs prior to TNF-α exposure was associated with a “negative z-score” (inhibition) (Figure 7b) and accompanied by the recovery of the “antioxidant action”.

**Figure 8:**
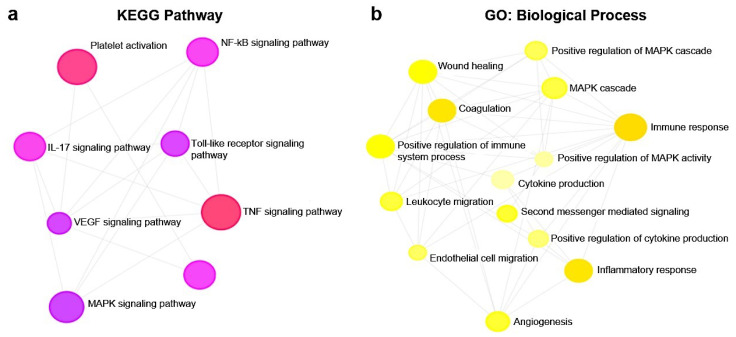
KEGG pathways involved in TNF-α response at vascular level (**a**) and GO (biological processes) (**b**) analyses were performed on 29 genes differentially expressed in EA.hy926 cells treated with TNF-α (20 ng/mL) alone and pretreated with B-ELNs (20 µg/mL) and then exposed to TNF-α (20 ng/mL).

Pathway enrichment analysis also revealed the modulation of a total of 121 KEGG pathways (Supplementary Table S3) and 121 GO biological processes (Supplementary Table S4). Among them, in Figure 8a we showed eight KEGG pathways (1.90 × 10^−10^ < *p*-value < 1.04 × 10^−4^) that resulted involved in NF-kB, TLR, TNF, MAPK, IL-17, VEGF signal pathways, leukocytes migration, and platelet activation modulated by our treatments. Interestingly, all these pathways where functionally correlated with several GO biological processes (9.98 × 10^−8^ < *p-*value < 9.32 × 10^−4^) involved in different endothelial functions (Figure 8b).

**Figure 9:**
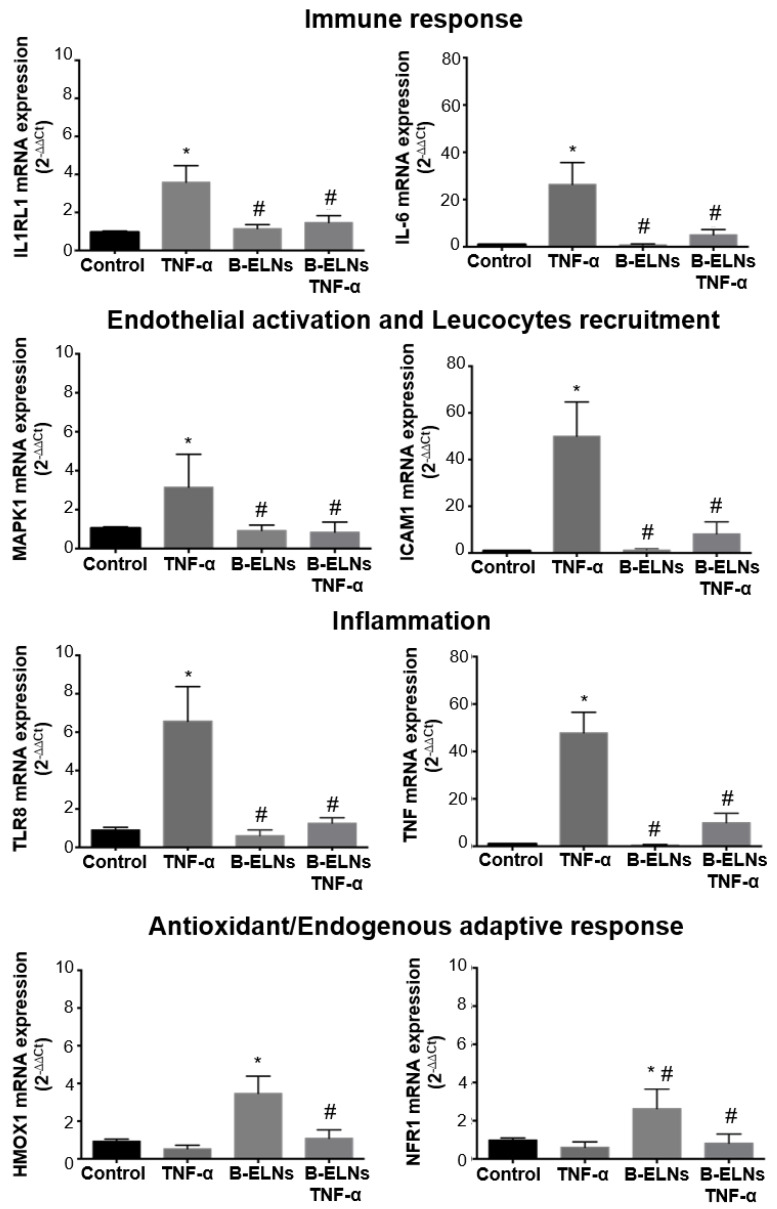
mRNA expression of selected DE genes by qRT-PCR. EA.hy926 cells pretreated for 3 h with B-ELNs (20 µg/mL) and then exposed for 2 h to TNF-α (20 ng/mL). Cells exposed to vehicle (PBS) only were used as a control. Values of mRNA expression are expressed as 2^−∆∆Ct^ normalized to control (n = 4). *p < 0.05 vs. control; # *p* < 0.05 vs. control treated with TNF-α.

Single qRT-PCR was performed on candidate genes selected among those belonging to the enriched GO categories and pathways (Figure 7 and Figure 8) arbitrarily cropped as follow: “Immune response” IL-6 and IL1RL1; “Endothelial activation and leucocytes recruitment” MAPK1 and ICAM; “Inflammation”, TLR8 and TNF; “Antioxidant and endogenous adaptive responses” HMOX1; and NRF1 (Figure 9), by picking one of them within the genes detected by TLDA card and selecting the second one according to our data interrogation.

### 3.5. miR-156e, miR-162, and miR-319d in B-ELNs Potentially Regulate Mammalian Inflammation-Related Genes

**Figure 10:**
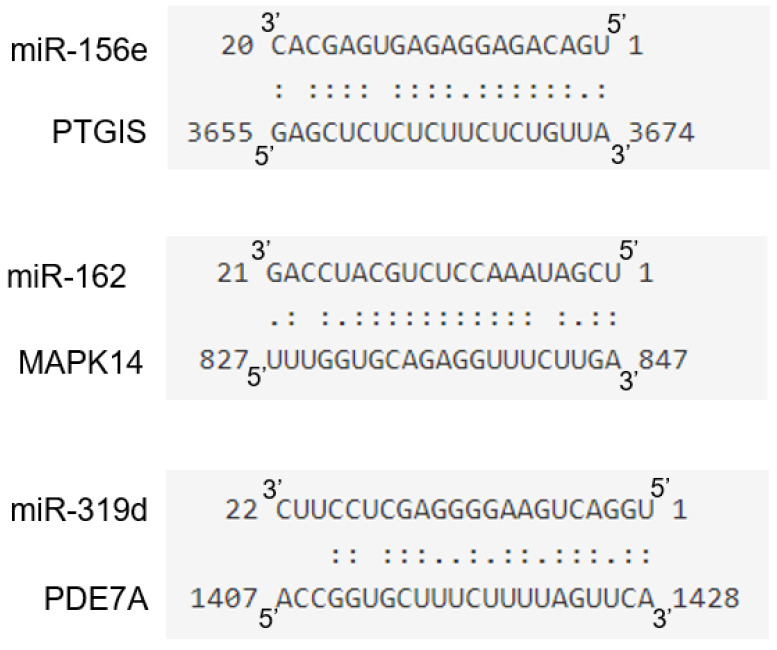
Diagram of putative miR-156e, miR-162, and miR-319d binding sites in PTGIS, MAPK14, and PDE7A, respectively. Paired bases are indicated by two vertical dots and a mismatch is indicated by one dot.

In order to identify potential molecular effectors able to mediate the observed functional effect of B-ELNs exposure on the transcriptional response to TNF-α we considered the set of miRNAs identified in B-ELNs by Xiao and coworkers [35]. Utilizing this miRNA set, a total of 226 targets were predicted by psRNATarget software (Supplementary Table S5). Among them, we searched for candidate mRNA targets belonging to the panel of DE genes that we found regulated by B-ELNs (Figure 6) and the genes involved in the pathways most significantly related to inflammatory response, cytokine release and oxidative stress (Figure 7 and Supplementary Table S2). The results of target gene prediction revealed that the following three miRNAs contained in B-ELNs, miR-156e, miR-162, and miR-319d, potentially target and theoretically regulate PTGIS, MAPK14, and PDE7A genes, respectively (Figure 10).

## 4. Discussion

ELNs have been identified in different edible plants [43] and fruits [5], although the exact cellular origin of these vesicles remains unclear. Recent observations have demonstrated the ability of these nanoparticles to carry bioactive compounds [43,44]. In addition, Ju S. et al. [11] reported that Grape ELNs can travel within the gut where they can be taken up by mouse intestinal stem cells.

Moreover, from certain fruits, of note lemons and grape [15,18], a cross-kingdom biological effects have been demonstrated, suggesting a possible therapeutic application of isolated fruit vesicles [4,45].

Here we investigated the presence of ELNs in blueberry fruit and to the best of our knowledge, this is the first study assessing the uptake of B-ELNs and their ability to induce a gene expression profile change in endothelial cells (EA.hy926).

Firstly, we isolated and characterized B-ELNs and we showed that they have exosome-like features (Figure 1). When we exposed EA.hy926 cells to different doses of B-ELNs we observed a dose-dependent uptake that resulted linear up to 6 h when 20 µg/mL of B-ELNs proteins dose was used (Figure 2a,b). Although the administration of a dose equal to 20 µg/mL B-ELNs (protein nanoparticles) to cells is likely to be not achievable in human, this methodological choice facilitated the comparison with previous studies addressing the effect of plant and fruit derived nanoparticles in mammalian tissues that utilized similar concentrations [11,16].

With the tested dose, B-ELNs did not affect cells viability per se. However, the exposure to B-ELNs prior to TNF-α treatment was able to decrease TNF-α-induced cell death in EA.hy926 cells (Figure 4). Several in vitro and in vivo studies have reported that TNF-α induces ROS generation and an imbalance of cellular redox status [46], both having an important role in determining cell damage and a commitment to apoptosis [47]. According to these observations, in our experimental conditions TNF- α treatment increased ROS generation, but this effect was counteracted by B-ELNs pretreatment (Figure 5).

In order to investigate the molecular mechanism behind this observation, and to identify a set of genes significantly modulated by TNF-α in EA.hy926 cells we analyzed inflammation-related genes using a TLDA “inflammatory” customized card. We identified 29 DE genes (fold change > 1.5) induced by TNF-α compared to control, that were modulated in the opposite direction when EA.hy926 cells were pretreated with 20 µg/mL B-ELNs (Figure 6), suggesting that such DE genes could be key factors for the B-ELNs-dependent reversion of a pro-inflammatory-like condition activated by TNF-α-exposure.

The pathway enrichment analysis revealed that among the 340 canonical pathways (Supplementary Table S2), there were some, modulated by pre-treatment with B-ELNs and TNF-α compared to TNF-α alone, potentially relevant in vascular functions and inflammation response, such as IL-6, Endothelin-1 and Leukocytes extravasation, TLR and NF-kB signaling pathways, and Antioxidant and Vitamin C signaling pathway (Figure 7). Furthermore, we found that TNF-α exposure was able to modulate a total of 121 KEGG pathways (Supplementary Table S3) and 121 GO biological process (Supplementary Table S4). In Figure 8 we reported selected KEGG pathway and GO biological process that might be responsible of TNF-α response on EA.hy926 cells and therefore of the ability of B-ELNs to reverse this effect.

Gene validation confirmed that EA.hy926 cells pretreatment with B-ELNs reversed the effect of TNF-α-induced mRNA expression of IL-6, IL1RL1, MAPK1, ICAM1, TRL8, and TNF and restored the antioxidant cellular power by the positive modulation of HMOX1 and NRF1 (Figure 9).

Our findings are in line with the ability of TNF-α to induce inflammatory response and interleukins release [48,49]; oxidative stress [47,50]; and MAPK activation [48,51] at a vascular level and bring to light new findings related to the field of natural compound and their possible use in the modulation of endothelial dysfunction induced by several stressor [22,23,52].

In particular, our results reveal that B-ELNs are able to modulate TNF-α response by promoting the increase of endogenous antioxidant defenses as well as other components in plants and fruits.

At this point our observation let an issue open: What mediated the observed functional effect of B-ELNs exposure? Referring to a paper previously published by Xia et al. [35] we considered the set of miRNAs in B-ELNs identified by the authors and searched for candidate mRNA targets within the panel of DE genes reported in our study. Intersecting their results with ours, we found three miRNAs contained in B-ELNs, such as miR-156e, miR-162, and miR-319d, that can be potentially responsible for the down-regulation of PTGIS, MAPK14 and different isoforms of PDE, modulating the response to TNF-α (Figure 10; Supplementary Table S5). These observations provide one of the possible mechanisms underlying the down-regulation by B-ELNs of those genes that had been up regulated by TNF-α exposure. Moreover, this finding agrees with the hypothesis raised by several investigators of the occurrence of a “cross kingdom” transfer of genetic material such as miRNAs through diet [9,10,53]. Further studies are necessary to confirm this hypothesis and possibly characterize the specific mediators of the observed effect.

Even though we focused our attention on miRNAs, we do not claim that miRNAs are the solely responsible of the observed effects on the modulation of TNF-α. It is in fact plausible that other cargo, including proteins or mRNA, may contribute to the observed effects. Nonetheless, miRNAs are plausible actors. Consequently, we can also hypothesize that the administration of vesicles either directly provides an exogenous source of bioactive molecules (miRNAs, proteins etc.) or stimulates the endogenous expression of any kind of response modulators including miRNAs.

## 5. Conclusions

Our findings provide support to our primarily hypothesis in which we affirm that ELNs in food item could represent a new class of “bioactive” food components, beside the already known class of phytochemical compounds.

In conclusion, our study reported for the first time the effect mediated by B-ENLs on endothelial cells challenged by TNF-α. The study, started from a “proof of principle” approach, that natural compounds have protective effect in several pathologic conditions, but here we demonstrated that ELNs in plants and fruits might represent new bioactive food components and therefore new “actors” in the biological observed effects. Future studies could be directed toward the use of ELNs as vectors to carry new active compounds such as RNAs, proteins and lipids.

## Supplementary Materials

The following are available online at https://www.mdpi.com/2218-273X/10/5/742/s1, Figure S1: Content of cyanidin-3-0-glucoside equivalent (C3G) in blueberry juice, Figure S2: Protein content of B-ELNs isolated from different BB juice. Bar represent protein amount (mg/mL) for each B-ELNs isolation, Table S1: List of genes and probes selected to undergo to real-time qPCR analysis, Table S2: Enrichment canonical pathways in EA.hy926 pretreated with B-ELNs the exposed to TNF-α, Table S3 KEGG pathways potentially altered by the 29 human genes that might be affected by B-ELNs (Referred to Figure 8a), Table S4: GO family (Biological Process) potentially altered by the 29 human genes that might be affected by B-ELNs (Referred to Figure 8b), Table S5: List of putative targets for selected miRNAs predicted by psRNATarget software.

## Abbreviations

ELN	exosome-like nanoparticles
EVs	extracellular vesicles
HMOX1	heme oxygenase (decycling) 1
IL-6	interleukin 6
ICAM-1	Intercellular Adhesion Molecule 1
IL1RL1	Interleukin 1 receptor-like 1
MAPK	Mitogen-activated protein kinase
NAC	N-acetyl-l-cysteine
NF-kB	nuclear factor kappa-light-chain-enhancer of activated B cells
NRF1	nuclear respiratory factor 1
PDE7A	phosphodiesterase 7A
PTGIS	prostaglandin I2 (prostacyclin) synthase
PTGS2	Prostaglandin-endoperoxide synthase 2/ cyclooxygenase-2
ROS	reactive oxygen species
TLR8	Toll-like receptor 8
TNF-α	tumor necrosis factor alpha
