# Supplementary material for: Blueberry-Derived Exosome-Like Nanoparticles Counter the Response to TNF-α-Induced Change on Gene Expression in EA.hy926 Cells
Source: Biomolecules. 2020 May 10;10(5):742. doi: 10.3390/biom10050742 (PMC7277966; doi:10.3390/biom10050742)
Supplement: Supplementary file 1 [file biomolecules-10-00742-s001.zip › Supplemental /Biomolecules-760980_Supplemental Material_Revised.docx]

**Supplemental Material**


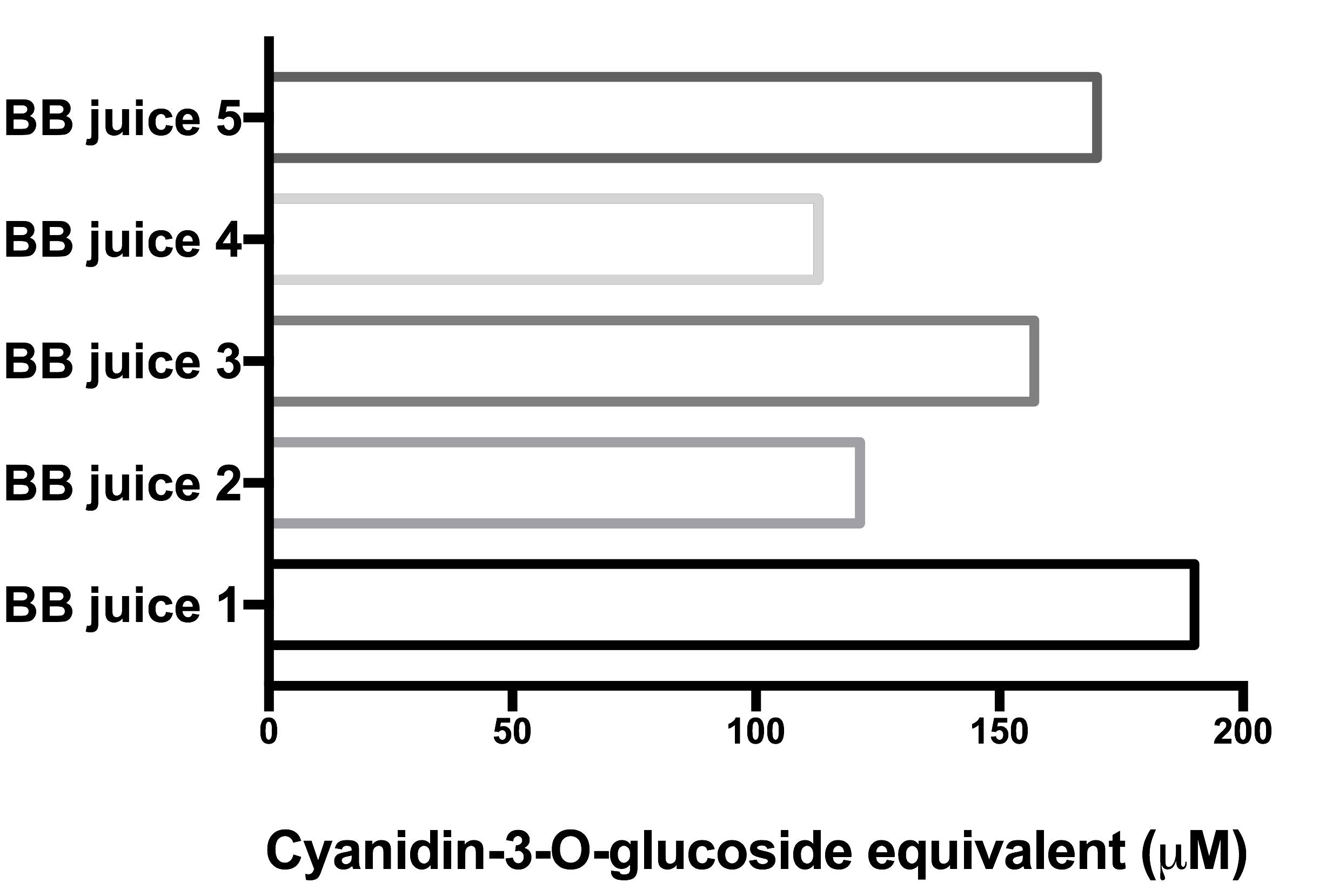


**Supplemental figure S1**. Content of cyanidin-3-0-glucoside equivalent (C3G), based on its specific absorbance peak at 520 nm in BB juice, measured before being processed for ELNs isolation. Bar represent concentration (μM) for each juice.

**Supplemental figure S2**. Protein content of B-ELNs isolated from different BB juice. Bar represent protein amount (mg/mL) for each B-ELNs isolation.

**Supplemental table S1**. List of genes and probes selected to undergo to real-time qPCR analysis.

| **Gene Symbol** | **Gene Name** | **Assay ID** |
| --- | --- | --- |
| *ACTB* | Actin | Hs01060665_g1 |
| *GUSB* | glucuronidase beta | Hs00939627_m1 |
| *HMOX1* | heme oxygenase 1 | Hs01110250_m1 |
| *ICAM1* | intercellular adhesion molecule 1 | Hs00164932_m1 |
| *IL1RL1* | interleukin 1 receptor like 1 | Hs00249384_m1 |
| *IL6* | interleukin 6 | Hs00174131_m1 |
| *MAPK1* | mitogen-activated protein kinase 1 | Hs01046830_m1 |
| *NRF1* | nuclear respiratory factor 1 | Hs00602161_m1 |
| *TLR8* | toll like receptor 8 | Hs00152972_m1 |
| *TNF* | tumor necrosis factor | Hs00174128_m1 |
