# Supplementary material for: Blueberry-Derived Exosome-Like Nanoparticles Counter the Response to TNF-α-Induced Change on Gene Expression in EA.hy926 Cells
Source: Biomolecules. 2020 May 10;10(5):742. doi: 10.3390/biom10050742 (PMC7277966; doi:10.3390/biom10050742)
Supplement: Supplementary file 1 [file biomolecules-10-00742-s001.zip › Supplemental /Biomolecules-760980_Supplemental Material_Revised.pdf]

Supplemental Material

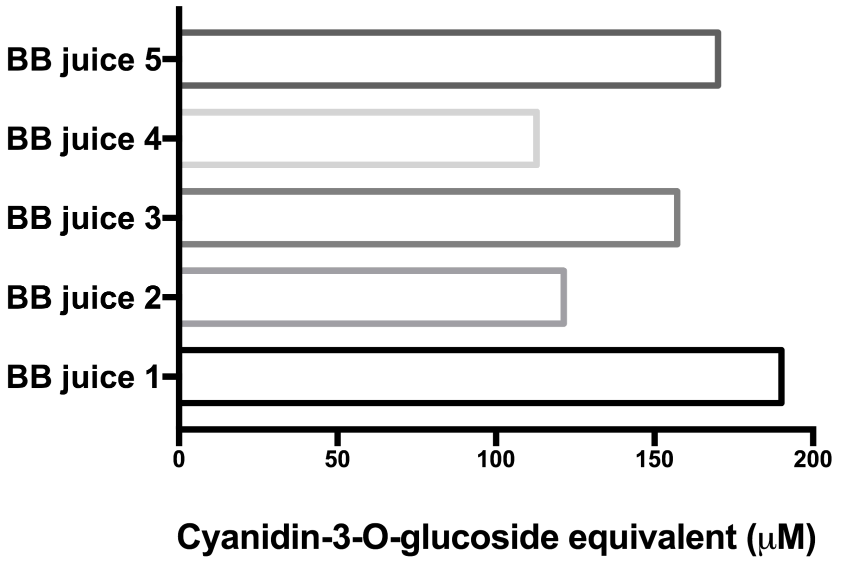

Supplemental figure S1. Content of cyanidin-3-0-glucoside equivalent (C3G), based on its specific absorbance peak at 520 nm in BB juice, measured before being processed for ELNs isolation. Bar represent concentration (μM) for each juice.

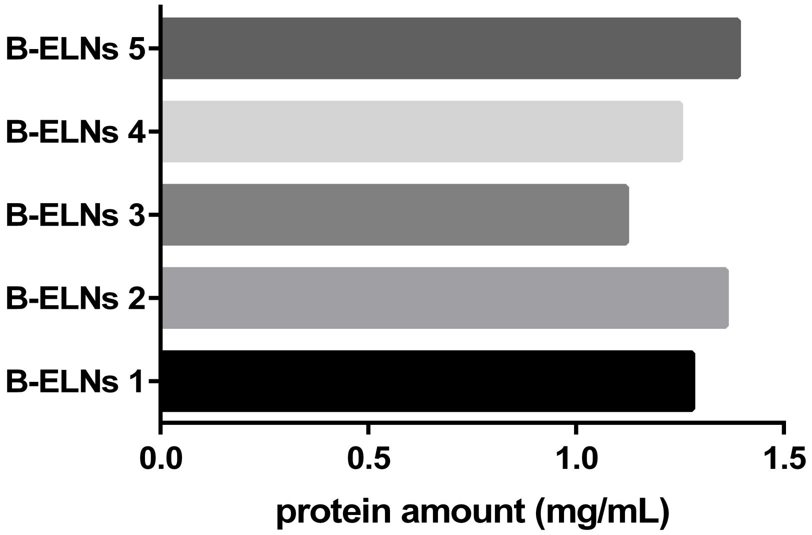

Supplemental figure S2. Protein content of B-ELNs isolated from different BB juice. Bar represent protein amount (mg/mL) for each B-ELNs isolation.

Supplemental table S1. List of genes and probes selected to undergo to real-time qPCR analysis.

Gene Symbol	Gene Name	Assay ID
ACTB	Actin	Hs01060665_g1
GUSB	glucuronidase beta	Hs00939627_m1
HMOX1	heme oxygenase 1	Hs01110250_m1
ICAM1	intercellular adhesion molecule 1	Hs00164932_m1
IL1RL1	interleukin 1 receptor like 1	Hs00249384_m1
IL6	interleukin 6	Hs00174131_m1
MAPK1	mitogen-activated protein kinase 1	Hs01046830_m1
NRF1	nuclear respiratory factor 1	Hs00602161_m1
TLR8	toll like receptor 8	Hs00152972_m1
TNF	tumor necrosis factor	Hs00174128_m1
